# Modification mapping by nanopore sequencing

**DOI:** 10.3389/fgene.2022.1037134

**Published:** 2022-10-28

**Authors:** Laura K. White, Jay R. Hesselberth

**Affiliations:** Department of Biochemistry and Molecular Genetics, RNA Bioscience Initiative, University of Colorado School of Medicine, Aurora, CO, United States

**Keywords:** RNA, modification, nanopore, sequencing, DNA

## Abstract

Next generation sequencing (NGS) has provided biologists with an unprecedented view into biological processes and their regulation over the past 2 decades, fueling a wave of development of high throughput methods based on short read DNA and RNA sequencing. For nucleic acid modifications, NGS has been coupled with immunoprecipitation, chemical treatment, enzymatic treatment, and/or the use of reverse transcriptase enzymes with fortuitous activities to enrich for and to identify covalent modifications of RNA and DNA. However, the majority of nucleic acid modifications lack commercial monoclonal antibodies, and mapping techniques that rely on chemical or enzymatic treatments to manipulate modification signatures add additional technical complexities to library preparation. Moreover, such approaches tend to be specific to a single class of RNA or DNA modification, and generate only indirect readouts of modification status. Third generation sequencing technologies such as the commercially available “long read” platforms from Pacific Biosciences and Oxford Nanopore Technologies are an attractive alternative for high throughput detection of nucleic acid modifications. While the former can indirectly sense modified nucleotides through changes in the kinetics of reverse transcription reactions, nanopore sequencing can in principle directly detect any nucleic acid modification that produces a signal distortion as the nucleic acid passes through a nanopore sensor embedded within a charged membrane. To date, more than a dozen endogenous DNA and RNA modifications have been interrogated by nanopore sequencing, as well as a number of synthetic nucleic acid modifications used in metabolic labeling, structure probing, and other emerging applications. This review is intended to introduce the reader to nanopore sequencing and key principles underlying its use in direct detection of nucleic acid modifications in unamplified DNA or RNA samples, and outline current approaches for detecting and quantifying nucleic acid modifications by nanopore sequencing. As this technology matures, we anticipate advances in both sequencing chemistry and analysis methods will lead to rapid improvements in the identification and quantification of these epigenetic marks.

## Overview of nanopore sequencing

**FIGURE 1:**
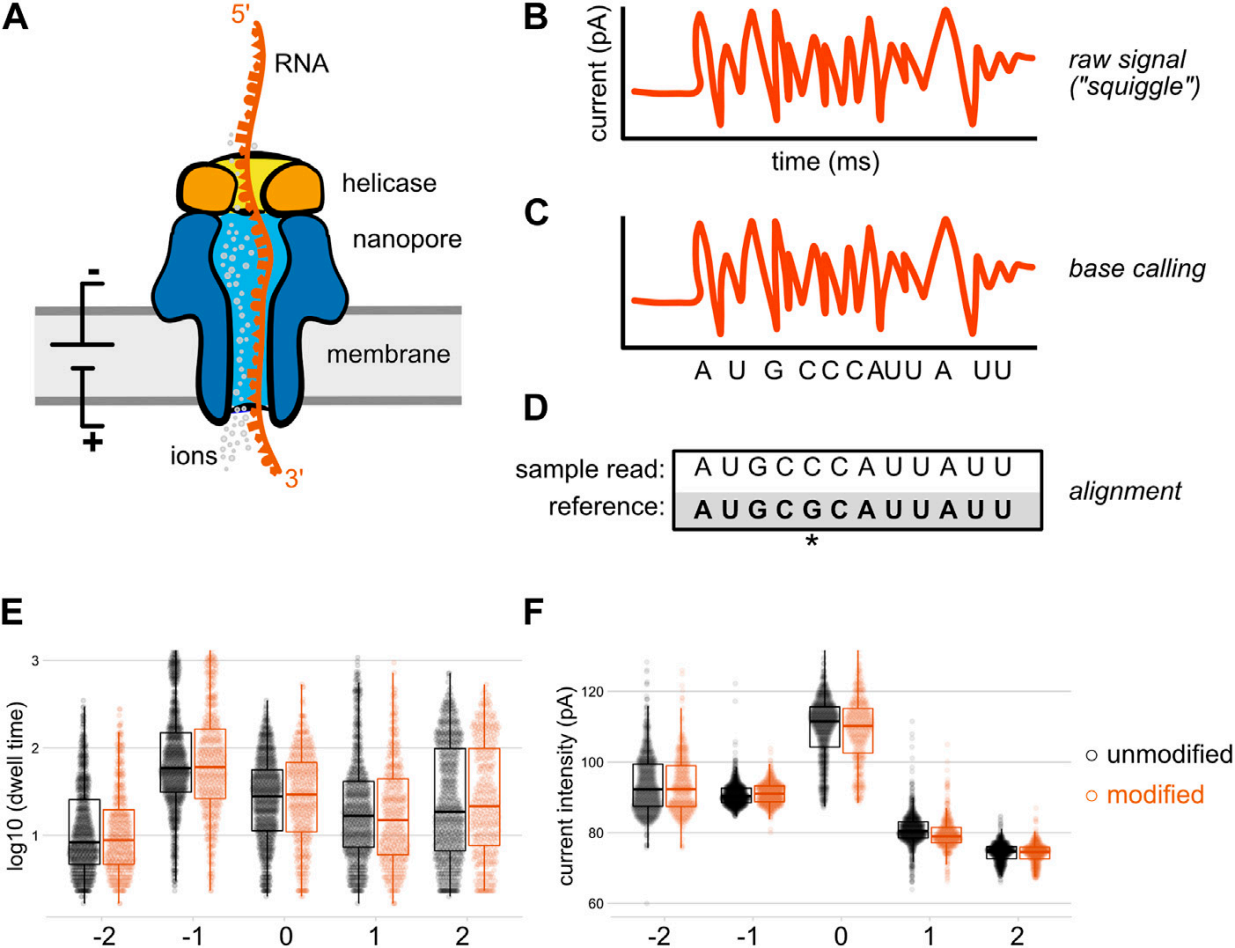
Direct RNA sequencing by nanopore. In direct RNA sequencing, the 3′-ends of single-stranded native RNA or the RNA strand of a cDNA/RNA hybrid are ligated to a sequencing adapter that has been pre-loaded with a helicase **(A)** When introduced into the nanopore flow cell, the helicase docks with one of thousands of individual nanopores embedded within a charged membrane, and threads its nucleic acid cargo into the central channel of the nanopore at an average speed of approximately 70 bases per second (Garalde et al., 2018). As the RNA molecule passes through a constriction point within the central channel of the nanopore (the “reader head”), changes in the flow of ions create local alterations in electric current signals that are sensed by an embedded ammeter. Individual nucleobases (depicted here as different shapes) impede the flow of ions to different degrees, producing characteristic signals in the nanopore raw output **(B)**, which is sometimes termed the “squiggle.” These raw signals are converted into sequence by a base calling algorithm **(C)** that compares the signal produced over a five nucleotide window within the center of the reader head to known signals produced by every possible five nucleotide RNA sequence **(D)** The base called data from each direct RNA read can then be aligned to a reference sequence; here, an aligned read with a mismatch to the reference at the fifth nucleotide is shown. Optionally, these aligned reads can later be re-annotated with raw signal information through a process called “resquiggling” (not depicted), enabling current intensity and dwell time signals to be associated with positional sequence information. Panels **(E)** and **(F)** depict our reanalysis of raw, resquiggled read signals for an m6A modification located at position 0 on a synthetic oligonucleotide, compared to an unmodified control (Leger et al., 2021). The differences in raw signal between modified and unmodified bases may be quite subtle compared to the signal differences between canonical nucleotides, and are best analyzed *via* machine learning algorithms and/or downstream analysis tools.

First conceptualized in the 1980s (Tobkes et al., 1985), nanopore sequencing uses a modified transmembrane protein (the nanopore) as both a channel through which a nucleic acid passes, and a biosensor capable of sensing the nucleobase content of that nucleic acid (Deamer et al., 2016). By embedding the nanopore within a membrane with a constant voltage bias, an ionic current drives single stranded nucleic acids through the pore (Clamer et al., 2014); at the narrowest aperture of this pore (the “reader head”), the flow of ions is differentially suppressed depending on the size and shape of the nucleobases present (Cherf et al., 2012; Manrao et al., 2012; Smith et al., 2015). While early proof of principle experiments used DNA polymerases to slow down this translocation process (Cherf et al., 2012; Manrao et al., 2012), the current commercial solution from Oxford Nanopore Technologies (ONT) employs an engineered helicase enzyme to both unwind double stranded molecules and introduce single stranded nucleic acid into the nanopore sensor at a controlled rate for sequencing (Figure 1A). However, these motor protein activities are stochastic, meaning that the time intervals between each stepwise advance of the DNA or RNA molecule are variable (Deamer et al., 2016), with current helicases averaging ∼70 bases per second for RNA (Garalde et al., 2018) and up to 450 bases per second for DNA when coupled with an R9.4 nanopore (Wang Y. et al., 2021).

In its current embodiment, the ONT MinION platform uses flow cells containing 2,048 individual nanopores divided across 512 active channels, each of which can be individually controlled by an application-specific integrated circuit. Samples are prepared for sequencing by ligating a sequencing adapter containing a pre-bound helicase enzyme to one end of genomic DNA, cDNA, or RNA molecules. When introduced into the flow cell, helicases and their nucleic acid cargo dock with these nanopores and ratchet the single stranded molecules into the pore; Figure 1 depicts this using a direct RNA sequencing read as an example. As the nucleic acid analyte passes through the narrowest point of the pore, it creates transient obstructions in the flow of ions through this constriction point, generating detectable changes in ionic current (Figure 1A). Importantly, the size and shape of this constriction point within the nanopore reader head means that multiple nucleotides contribute to the ionic current signals produced as a nucleic acid passes through the pore (Laszlo et al., 2014). More recently, ONT has released R10 and R10.3 nanopores that contain two such constriction points, in an effort to gain higher accuracy when calling homopolymeric stretches of nucleotides (Tytgat et al., 2020; Huang Y.-T. et al., 2021).

Nanopore sequencing produces a raw output of current intensity over time, measured in picoamps and milliseconds, respectively (Figure 1B). Sometimes referred to as “the squiggle” in ONT parlance, this raw signal can be base called in real time during a sequencing run by the MinKNOW software that controls the sequencing device. The output of this optional local base calling step is stored as a fastq file, and current signal over time as well as metadata about the sequencing run are stored in binary HDF5 file format known as a FAST5 file (A recent community development effort has proposed an alternative file format, SLOW5, which permits 25% smaller output files and 15–30 fold more efficient analysis on high performance computing systems (Gamaarachchi et al., 2022), but whether this will replace FAST5 as the standard format for raw nanopore data remains to be seen.) If base calling has not been performed during the sequencing run, or if higher accuracy is desired, this data is then used as an input for base calling, the process by which raw current signal is converted into read sequence (Figure 1C) before subsequent alignment to one or more reference sequences (Figure 1D).

Before discussing how nanopore sequencing has been used to detect nucleic acid modifications, it is useful to understand the general principles by which raw ion current signal information is converted into sequence data by a base calling. A number of base calling software tools have been developed by both ONT and the research community (Timp et al., 2012; Boža et al., 2017; David et al., 2017; Stoiber and Brown, 2017; Teng et al., 2018; Wick et al., 2019; Zeng et al., 2019), and improvements in base calling algorithms have been major contributors to increases in raw read sequencing accuracy from ∼85% when commercial nanopore sequencing was first introduced to present accuracy estimates of 99.6% when signal from a R10.4 chemistry DNA sequencing run is base called using the current “official” ONT base caller, Guppy, in “super accuracy” mode (Accuracy). For direct RNA sequencing, which still relies on the previous R9.4 pore chemistry and uses a separate base calling model, raw read accuracy rates have trailed those of DNA sequencing by approximately 5% (Soneson et al., 2019; Delahaye and Nicolas, 2021), and the expected throughput for direct RNA sequencing is roughly an order of magnitude lower than if the same samples were converted to PCR-amplified cDNA.

The Guppy basecaller uses a recurrent neural network (RNN) to associate raw signals contained within the FAST5 file with known signals from a training set containing probable signal distributions for all possible k-mers. Guppy’s current base calling algorithms have been trained on a range of DNA and RNA sequencing data to be able to predict sequence based on the current changes as the nanopore reader head interrogates a k nucleotide window (five nucleotides for RNA, and six for DNA). Once an appropriate match is identified, the central nucleotide of this k-mer is added to a fastq file containing the sequencing read (Wan et al., 2022). Each called base is also accompanied by a quality score that captures the base calling algorithm’s predicted confidence in the nucleobase assignment. Because the RNN is bi-directional, these predictions are informed by the ion current signatures produced both earlier and later in the sequencing read, generating improved prediction accuracy over previous base calling modalities (Wan et al., 2022). Guppy contains various models that can be used for real-time base calling (at lower accuracy) or higher accuracy offline base calling of DNA or RNA, all of which were initially trained on unmodified nucleic acids.

## Bioinformatic strategies for *de novo* modification detection

DNA and RNA modifications can produce changes in both current and translocation time as the modified base transits through a nanopore. To detect these modifications at the base calling level, machine learning algorithms must be trained on data containing both modified and unmodified bases in many different sequence contexts. In recent years, ONT has developed DNA base calling models within Guppy using training data for m6A and m5C modifications; however, integrating modification detection into the base calling step reportedly reduces in base calling accuracy. To address this issue, the ONT-developed tool Remora identifies modified bases using a separate algorithm which is run immediately following canonical Guppy base calling, thereby separating the base calling and modification detection steps. The public release of this tool allows researchers to train models for the prediction of other modified bases. ONT has also integrated Remora’s 5mC detection into the MinKNOW sequencing software, enabling less sophisticated users to detect cytosine methylation at CpG sites in parallel with their sequencing run. Future development efforts by both ONT and the research community to train Remora on other modified nucleotides are expected to expand the catalog of base modifications that can be detected in concert with base calling.

Despite these advances, several major obstacles remain to detecting a broad repertoire of nucleic acid modifications by machine learning approaches. Developing robust algorithms for modified base calling requires DNA and RNA training sets that contain modified nucleotides in all possible sequence contexts, a non-trivial endeavor for nucleic acid modifications that cannot be readily generated by chemical synthesis or enzymatic modification (Begik et al., 2022). This includes the majority of the 170+ endogenous modifications present on RNA molecules (Boccaletto et al., 2022). Moreover, current algorithms cannot be used to call multiple types of epigenetic modifications simultaneously; instead, an algorithm for each specific modification must be selected and applied individually. For modifications whose position has been experimentally validated *via* an orthogonal method over a wide variety of sequence contexts, it is possible to use a supervised learning approach to train existing base callers or Remora models to detect a specific DNA or RNA modification in nanopore data (Stoiber et al., 2017; Lee et al., 2020). For all other modifications, alternative strategies are required (Furlan et al., 2021). As a thorough review of machine learning approaches for combining base calling and modification detection has been recently released by Wan and others (Wan et al., 2022), this review primarily focuses on approaches to detecting the majority of nucleic acid modifications where insufficient training data exists to enable their identification in parallel with base calling.

**FIGURE 2:**
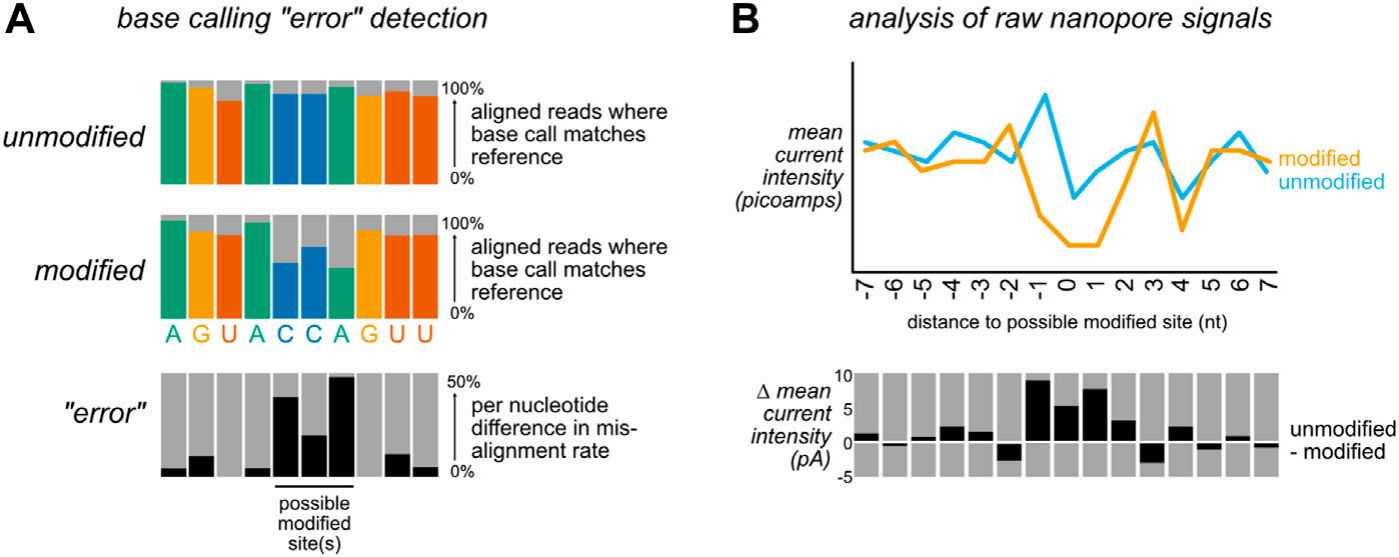
Bioinformatic approaches for detecting nucleic acid modifications. To detect modifications that are not included in existing base calling algorithm models, most bioinformatic tools take one of two non mutually exclusive strategies: **(A)** After alignment to a reference sequence, differences in alignment rates can be compared between a modified and unmodified (or control vs knockdown) sample. Sites with a high difference in mismatch, insertion, deletion, and/or base calling quality score (sometimes referred to as “trace”) between the two samples are identified as candidate modified positions. Here, differences in mismatch percentage are illustrated. **(B)** After alignment to a reference sequence, the aligned reads are re-annotated (“resquiggled”) with the raw nanopore signal information, enabling per-nucleotide examination of both current intensity and the duration of time that an individual nucleotide spent within the nanopore (“dwell time”). These features can either be analyzed in aggregate (as in the depiction of mean current intensity above), or on a per-read basis.

In general, bioinformatic approaches for detecting modifications in nanopore data in the absence of a modification-specific base calling model take one of two approaches. First, examining the consensus accuracy of base calling after alignment can enable identification of modification-specific base calling errors in the form of increased mismatches, insertions, or deletions at modified sites, as well as decreases in the base caller’s confidence in calling a nucleotide (Figure 2A). Although these are typically referred to as “error” focused methods, the errors in question are expected to be reflective of biology rather than the result of base calling inaccuracy *per se*. While these methods do not permit single-molecule level resolution of modifications, this approach can be quite powerful when comparing modified and unmodified samples. Sites with statistically significant differences between a modified and unmodified (or control vs knockdown) sample are identified as candidate modified sites for further analysis and/or validation. A second approach (Figure 2B) relies on the analysis of raw data features after reannotation of the raw data to incorporate alignment information (a step termed segmentation or “resquiggling”). This information can then be analyzed over all aligned reads at a given site, or on a single molecule basis. These two strategies are not mutually exclusive and in fact are often employed in tandem, as comparison of outputs from distinct tools can help compensate for the limitations of an individual approach, and may aid in identifying false positives and/or false negatives.

## Early detection of nucleic acid modifications by nanopore sequencing—lessons from DNA methylation

The cytosine nucleobase is a common substrate for epigenetic modification in DNA, with functional links to both development and disease in eukaryotes (Johnson et al., 2012; Akhavan-Niaki and Samadani, 2013; Smith and Meissner, 2013). As some of the first nucleic acid modifications to be detected *via* nanopore sequencing, cytosine methylation provides a useful illustration of the technical challenges and biological relevance of discriminating specific nucleic acid modifications. Improvements in sequencing technology, development and benchmarking of multiple bioinformatic approaches for detecting methylated cytosines in nanopore data, and the generation of modified and unmodified data sets for training modified base calling models have led to iterative refinement in predicting these modifications.

**FIGURE 3:**
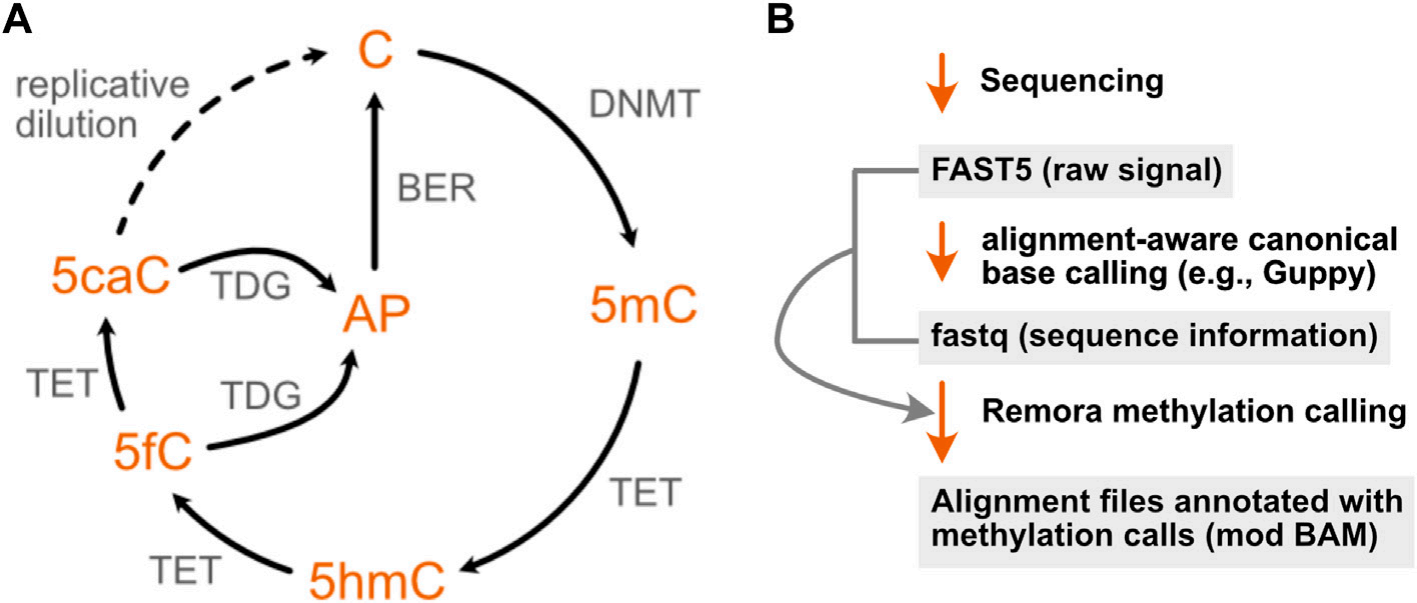
The cytosine methylation pathway has been the focus of extensive development of nanopore modification detection methods. **(A)** The methylation of cytosine by DNA methyltransferases (DNMTs) can be progressively reversed in a series of oxidation steps by ten-eleven translocase (TET) enzymes, generating 5-hydroxymethyl- (5hmC), 5-formyl- (5fC), and 5-carboxyl- (5caC) cytosine. The latter two modifications may each be converted to an abasic site (AP) by a uracil glycosylase family member (TDG), enabling repair by the base excision machinery (BER) to restore the original unmodified cytosine. A second, indirect mode of demethylation is through dilution of these epigenetic marks over multiple replication cycles, denoted by the dotted arrow. **(B)** Recent ONT software updates enable cytosine methylations to be identified using a lightweight base calling algorithm (Remora) that uses the output of canonical nanopore basecalling to identify 5mC and 5hmC modifications.

DNA methyltransferases (DNMTs) methylate genomic cytosine at the C5 position to form 5-methylcytosine (5mC), an epigenetic mark that leads to alterations in chromatin structure and gene silencing, largely at cytosine-guanine dinucleotide (CpG) motifs (Raiber et al., 2017). Altered patterns of cytosine methylation have been linked to disease, particularly cancer, where hypermethylation of tumor suppressors and/or hypomethylation of oncogenic factors can lead to inappropriate proliferation and dysregulated growth (Skvortsova et al., 2019). 5mC is also dynamically reprogrammable, and the reversal of this epigenetic mark is a stepwise process that generates several other DNA modifications (Figure 3A): ten-eleven translocation (TET) enzymes sequentially oxidize 5 mC to form 5-hydroxymethylcytosine (5hmC) and 5-formylcytosine (5fC), which may then be further oxidized to 5-carboxylcytosine (5caC) (He et al., 2011; Ito et al., 2011). Both 5 fC and 5caC can be converted to an abasic site (AP) by a uracil glycosylase family member (TDG), which is then repaired by the base excision repair machinery, restoring the site to an unmethylated cytosine (Jacobs and Schär, 2012).

Until recently, bisulfite sequencing, in which unmethylated cytosine is chemically converted to uracil (Frommer et al., 1992), has remained the gold standard for high throughput genome-wide mapping of cytosine methylation, despite the fact that this technique cannot distinguish between 5mC and 5hmC without additional chemical or enzymatic pre-treatment (Huang et al., 2010; Booth et al., 2012; Yu et al., 2012). The ability of nanopore sequencing to discriminate between methylated and unmethylated cytosine was first recognised in 2009, when Clarke and others reported the ability to distinguish cytosine monophosphates from 5mC using a mutant haemolysin nanopore (Clarke et al., 2009). A second report extended this finding to differentiate between 5mC, 5hmC, and unmethylated cytosine in single stranded DNA the following year (Wallace et al., 2010), and in 2014, Mark Akeson’s group at UCSC reported the ability to further identify distinct ionic current states of 5fC and 5caC, providing proof of principle for the discrimination of all epigenetic variants produced during C5 cytosine methylation using commercially available nanopore sequencing (Wescoe et al., 2014).

Three years later, simultaneous publications from the Timp laboratory and the UCSC Nanopore Group described the applicability of the ONT MinION platform for genome wide detection of DNA methylation by training machine learning algorithms to identify the ionic current distributions associated with known C vs. 5 mC sites (Rand et al., 2017; Simpson et al., 2017). Rand and others extended this observation to mapping 5mC, 5hmC and 6-methyladenosine (m6A) sites, and validated their results against 5mC locations previously identified using bisulfite sequencing (Kahramanoglou et al., 2012), reporting a classification accuracy of 80% for methylated cytosines on synthetic DNA. This approach leveraged the existence of bisulfite sequencing data to identify the ionic current distributions associated with known C, 5mC, and 5hmC sites. An initial model, termed signalAlign, was later incorporated into the software tool Nanopolish (Jain et al., 2018), enabling supervised learning approaches for other nucleic acid modifications. Further refinements of 5mC and 5hmC models have enabled both of these DNA modifications to now be incorporated into the ONT Guppy base caller and in the recent release of a new modification calling algorithm, Remora (Figure 3B), both of which permit the identification of modified vs unmodified bases in parallel with base calling. However, a comprehensive benchmarking of six computational approaches for 5mC detection in 2021, including Nanopolish- and Guppy-based pipelines, revealed a wide range of predicted methylation frequencies as well as tradeoffs in specificity and sensitivity across different tools, suggesting that Guppy base calling may not fully capture cytosine methylation, and that the comparison of results across multiple algorithms and downstream analysis tools may remain useful in identifying DNA methylation sites by nanopore sequencing (Yuen et al., 2021).

### Detection of other endogenous DNA modifications

Since the first genome-wide report of DNA methylation detection by MinION sequencing, a number of other intrinsic DNA modifications have also been identified in nanopore data at varying levels of scale and throughput (Table 1). The DNAmod database lists 41 naturally-occurring DNA modifications that have been cataloged across all kingdoms of life, 18 of which are exclusively produced *via* DNA damage (Sood et al., 2019). While these adducts may be tractable for nanopore sequencing, the stochastic nature of DNA damage and/or low stoichiometry of site-specific modification may prove challenging for their detection. Of the remaining 23 endogenous modifications, we have already discussed 5mC and demethylation derivatives, as well as m6A modifications. Aside from these, only a handful of the remaining 19 have been analyzed by nanopore sequencing. 8-oxo-guanine, a product of DNA oxidative damage that may also serve as an epigenetic mark, has been evaluated in a low throughput alpha-hemolysin nanopore assay but not yet by ONT sequencing (Liu et al., 2016). N4-methylcytosine (m4C), a common modification deposited by prokaryotic methylation-restriction systems (Weigele and Raleigh, 2016), has been mapped at its known sequence motifs in multiple bacterial species by nanopore sequencing (Tourancheau et al., 2021). Another nucleic acid modification deposited during interspecies conflict, the viral hypermethylation 7-cyano-7-deazaguanine (preQ0), has also been detected *via* comparison of raw nanopore signals derived from genomic DNA sequencing of wild-type and methylation mutants (Kot et al., 2020). Moreover, the genomes of several *S. aureus* jumbo bacteriophages that are presumed to be enriched for uracil-substituted DNA were sequenced on an ONT MinION but produced “sequence data that could not be interpreted” by Korn and others, suggesting that 5-hydroxymethyluracil and/or other deoxyuracil derivatives may produce detectable distortions in nanopore sequencing; the same samples were not sequenceable by standard short read methods until the authors prepared sequencing libraries using the uracil-insensitive polymerase PhusionU (Korn et al., 2021). Finally, the Nookaew laboratory used nanopore sequencing for the detection of phosphorothioate (PS) linkages (Wadley et al., 2022), which occur naturally in prokaryotes and archaea (Wang et al., 2007) and are also commonly used as stabilizing linkages during oligonucleotide synthesis to protect against nuclease digestion (Vosberg and Eckstein, 1982). Using their previously developed ELIGOS software tool, which identifies sites with high base calling error rates *via* comparative statistical tests between two samples (Jenjaroenpun et al., 2021), they identified sites with statistically significant differences in base calling error rates between PS+ and PS- samples, extracted raw nanopore features with Nanopolish, and analyzed those features to determine PS signatures in *Salmonella enterica* genomes, noting that ionic current disturbances occur at known sites of PS linkages. This study represents the first use of high throughput nanopore sequencing to detect modifications of the nucleic acid backbone (Wadley et al., 2022).

**TABLE 1:** Naturally-occurring and synthetic DNA and RNA modifications identified by nanopore sequencing. Modifications whose abbreviations are marked with an asterisk (*) have proof of principle data for detection in protein nanopore experiments, but lack detection evidence in commercial ONT sequencing at the time of this writing.

Molecule	Modification	Abbreviation	Origin	Select Publications	PMID
DNA	N6-methyladenosine	m6A	Naturally occuring	Rand et at. (2017)	28218897
DNA	5-methylcytosine	5mC	Naturally occuring	Simpson et at. (2017); Rand et at. (2017)	28218898, 28218897
DNA	5-hydroxymethylcytosine	5hmC	Naturally occuring	Rand et at. (2017)	28218897
DNA	5-formylcytosine	5fC*	Naturally occuring	Wescoe et at. (2014)	25347819
DNA	5-carboxylcytosine	5caC*	Naturally occuring	Wescoe et at. (2014)	25347819
DNA	N4-methylcytosine	m4C	Naturally occuring	Tourancheau et at. (2021)	33820988
DNA	8-oxo-7,8-dihydroguanine	8oxoG*	Naturally occuring	An et al. (2015)	25768204
DNA	7-cyano-7-deazaguanine	preQO	Naturally occuring	Kot et at. (2020)	32941607
DNA	chlorodeoxyuridine	CIdU	Synthetic	Georgieva et al. (2020)	32710620
DNA	5-bromo-2-deoxyuridine	BrdU	Synthetic	Muller et at (2019); Georgieva et al. (2020)	31011185, 32710620
DNA	iododeoxyuridine	IdU	Synthetic	Georgieva et al. (2020)	32710620
DNA	5-ethyny1-2-deoxyuridine	EdU	Synthetic	Georgieva et al. (2020)	32710620
DNA	biotin-dU	n/a	Synthetic	Georgieva et al. (2020)	32710620
DNA	phosphorothioate	PS	Synthetic	Wadley et at. (2022)	35531280
RNA	5-ethynyluridine	5EU	Synthetic	Maier et at. (2020)	32887688
RNA	5-bromouridine	5BrU	Synthetic	Maier et at. (2020)	32887688
RNA	5-iodouridine	51U	Synthetic	Maier et at. (2020)	32887688
RNA	4-thiouridine	4SU	Synthetic	Maier et at. (2020)	32887688
RNA	6-thioguanine	6SG	Synthetic	Maier et at. (2020)	32887688
RNA	2"-hydroxyl acylation	n/a	Synthetic	Aw et al. (2021); Stephenson et at. (2022)	33106685, 35252946
RNA	N6-methyladenosine	m6A	Naturally occuring	Garalde et al. (2018)	29334379
RNA	pseudouridine	111	Naturally occuring	Begik et at. (2021); Leger et at. (2021); Smith et al. (2019)	31095620, 33986546, 34893601
RNA	2"-0-methylation	2-0-Me	Naturally occuring	Stephenson et at. (2022); Begik et at. (2021); Leger et at. (2021)	33986546, 35252946, 34893601
RNA	N6,N6-dimethyladenosine	m6,2A	Naturally occuring	Leger et at. (2021)	34893601
RNA	5-methylcytosine	5mC	Naturally occuring	Garalde et al. (2018)	29334379
RNA	1-methylguanosine	m1G	Naturally occuring	Leger et at. (2021)	34893601
RNA	inosine	I	Naturally occuring	Leger et at. (2021)	34893601, 31740818
RNA	7-methylguanosine	m7G	Naturally occuring	Smith et al. (2019); Leger et at. (2021)	31095620, 34893601

## Detection of synthetic nucleic acid modifications

Another class of modifications that have been evaluated in nanopore sequencing are DNA and RNA modifications that do not occur naturally; these are also detailed in Table 1. The majority of these adducts are produced by chemical treatment of nucleic acids for one of three purposes: 1) to metabolically label newly synthesized RNA or DNA, 2) to interrogate accessibility of individual nucleotides, or 3) to manipulate an existing modification to alter and/or enhance the signal produced.

In the first category, a number of deoxyuridine derivatives are efficiently incorporated in place of thymine (a.k.a. 5-methyl-2′-deoxyuridine), and can be added in a pulsed dose to label newly synthesized DNA. These analogs include 5-bromodeoxyuridine (BrdU) and 5-iodo-2′-deoxyuridine (IdU), which can both be recognized by commercial antibodies (Gratzner, 1982), and 5-ethynyl-2′-deoxyuridine (EdU), whose terminal alkyne group makes this labeling reagent amenable to click chemistry (Salic and Mitchison, 2008). Following a successful report of BrdU detection by nanopore sequencing in budding yeast (Müller et al., 2019), Georgieva and others tested 11 such thymidine analogs for use in labeling replicating or repaired DNA, demonstrating the MinION platform’s capacity for distinguishing all 11 analogs vs thymidine, with the strongest differential signals in ionic current produced by IdU, CldU, and biotin-dU (NB: Per communication with Oxford Nanopore applications scientists and based on our experience, sequencing of highly biotinylated analytes such as those incorporated *via* addition of biotin-NTPs is not recommended for high throughput nanopore sequencing applications due to the tendency to prematurely clog the flow cell pores, reducing sequencing yield.) While most signal distortions were clustered within 2-4 nucleotides from the DNA modification, this panel of modifications covered a spread of molecular weights from 242–1851 g/mol, with more structurally bulky adducts generating signal distortions across a larger footprint of nucleotides, up to 10 nucleotides from the modified base (Georgieva et al., 2020) (Based on previous reports of longer durations for heavier molecules in a single α-hemolysin nanopore (Robertson et al., 2007), they further reasoned that these larger adducts may have a longer dwell time in the pore.) Similarly to the strategy described above for DNA, the EdU ribonucleotide analog 5-ethynyluridine (5EU) can be used to pulse label nascent transcription, as well as to tag these RNA products *via* a click reaction. Maier and others leveraged this reagent in developing a technique called nano-ID, beginning by benchmarking signals from 5EU, 5-bromouridine (5BrU), 5-iodouridine (5IU), 4-thiouridine (4SU) and 6-thioguanine (6SG) on synthetic oligoribonucleotides, which demonstrated that 5EU and 5IU produce the largest changes in base calling error over the 5mer surrounding the nucleotide analog, while 5EU produces the most significant changes in ion current signal. Based on this comparison, they optimized a 5EU labeling protocol for the metabolic labeling of individual RNA isoforms, and showed that this technique can be used to measure isoform stability over time (Maier et al., 2020).

NGS workflows to interrogate DNA and RNA’s accessibility to labeling reagents have also begun to be adapted for nanopore sequencing. To measure chromatin accessibility, Shipony and others used EcoGII, an m6A methyltransferase with low sequence specificity, to methylate deoxyadenosine in open chromatin regions in a protocol called SMAC-seq (Shipony et al., 2020), inspired by previous Illumina short read sequencing techniques that interrogated chromatin accessibility using CpG and GpC 5mC methyltransferases (Kelly et al., 2012; Nabilsi et al., 2014; Krebs et al., 2017). They further showed that these three enzymes can be used in concert, which may be useful in species with high levels of genomic m6A methylation. Another recently reported technique, DiMeLo-seq, also uses a nonspecific m6A methyltransferase, but targets this methylation reaction by fusing the methyltransferase to an antibody against the centromeric histone protein CENP-A, enabling both enrichment of CENP-A enriched chromatin by immunopurification, and also the detection of proximal methylated sites (Altemose et al., 2022). Both of these methods are tractable for analysis using bioinformatic pipelines developed for detecting endogenously deposited DNA methyl marks.

In contrast to the DNA accessibility approaches described above, methods for interrogating the accessibility of RNA by high throughput sequencing largely rely on chemical reagents that produce non-natural RNA adducts (Siegfried et al., 2014; Wang X.-W. et al., 2021). To date, only a few publications describe the adaptation of these approaches to nanopore direct RNA sequencing. Aw and others tested five chemical structure probes, including DMS and SHAPE reagents, on a *Tetrahymena* RNA with well characterized structure (Aw et al., 2021). While DMS-probed samples produced large base calling errors, evaluation of the predictive power of all five reagents against known sites from footprinting gel data revealed that treatment with the RNA acylation reagent NAI-N3 produced the most useful base calling error signatures for detection of RNA structure. Based on this analysis, the authors used Nanopolish (Loman et al., 2015) to resquiggle their data, extracting raw signal features and training a model to detect these adducts. After examining the reactive sites, they concluded that NAI-N3 treatment produces base calling errors and raw current changes at modified nucleotides, but does not produce statistically significant changes in dwell time within a 5-mer window surrounding the modified positions. In contrast, a recent study from Stephenson et al. also examined the impact of RNA acylating SHAPE reagents (in particular, acetylimidazole) on raw nanopore signal in direct RNA sequencing, but extended their analysis over a wider window of signal in order to capture transient translocation slowdowns due to interactions between RNA adducts and the nanopore helicase, demonstrating that RNA acylation produces alterations in both current and time features in raw nanopore data (Stephenson et al., 2022).

## Direct detection of endogenous RNA modifications

Direct RNA sequencing by nanopore is currently unique in its capacity to sequence native RNA molecules without the need for reverse transcription or PCR amplification steps, making it particularly well suited to the study of RNA modifications. This was appreciated from the first preprint report of direct RNA sequencing on the MinION platform in 2016, wherein Garalde and others demonstrated distinct current signals for m6A and m5C modified RNA on *in vitro* transcribed and fully-modified RNA standards (Garalde et al., 2018). m6A is the most common RNA modification, and the tools and approaches to detect and quantify this adduct have multiplied rapidly, with at least four m6A-specific analysis tools available for RNA at the time of this writing (Hendra et al., 2021; Liu et al., 2019; Lorenz et al., 2020; Gao et al., 2021; Pelizzola et al., 2021), and a range of others capable of analyzing m6A or other signals for feature differences across two or more comparative samples (Furlan et al., 2021; Wan et al., 2022).

These same approaches have also been applied to the detection of other naturally occurring RNA marks. In addition to 5 mC and m6A (mentioned above), at least seven other classes of endogenous RNA modifications have been profiled by direct RNA sequencing. In 2019, Smith et al. published a report of nanopore sequencing of *E. coli* 16 S rRNA, in which both known 7-methylguanosine (m7G) and pseudouridine (Ψ) positions produced mismatch and current intensity deviations when compared to an unmodified sample (Smith et al., 2019). One limitation of pseudouridine mapping is that the base calling error and raw signal distortions produced by pseudouridines may be indistinguishable from those of N1-methylpseudouridines (Fleming and Burrows, 2022). In addition to analyzing pseudouridine sites, Begik and others described a collection of base calling error, ionic current, and base calling quality score (a.k.a. “trace”) signals produced at 2′-O-methyl (Nm) sites in *S. cerevisiae* rRNA, but note that the signals produced by 2′-O-methylation are less reproducible across different sequence contexts (Begik et al., 2021). A similar detection approach has also been used to identify inducible *Ψ* positions in interferon responsive genes (Huang S. et al., 2021), and a third pseudoU detection pipeline, Penguin, claims ∼93% accuracy in *Ψ* detection on mRNAs from HEK293 cells (Hassan et al., 2022). Stephenson and others also profiled Nm sites on yeast rRNA, noting that like RNA acylation, methylation of the 2′-hydroxyl position on RNA produces increases in dwell time at a registration distance consistent with interaction(s) with the nanopore motor protein (Stephenson et al., 2022). Similarly, our own work demonstrates that 2′-phosphates deposited during RNA ligation produce offset increases in dwell time consistent with helicase interactions, as well as alterations in current intensity and base calling errors (White et al., 2022). Several of the above modifications were also examined by Leger et al., who developed the Nanocompore pipeline for comparative modification detection in direct RNA sequencing, and validated this pipeline on synthetic oligos containing m6A, m5C, Ψ, 2′-O-methyladenosine, 1-methylguanosine (m1G), N6,N6-dimethyladenosine (m6,2A), and the naturally occurring purine nucleotide inosine (I), as well as RNA from *E. coli* with a knockout for an m7G-depositing methyltransferase (Leger et al., 2021). Putative A-to-G miscalls at inosine sites were described earlier by Workman and others (Workman et al., 2019), and Vo and others have also detected differential current signals at poly(I) tails produced *via in vitro* 3′-polyinosylation of RNA by the *Schizosaccharomyces pombe* nucleotidyl transferase Cid1 (Vo et al., 2021).

## Emerging areas in nanopore modification analysis

While m6A detection by direct RNA sequencing is not yet routine, the focus has shifted from proof of principle that m6A RNA modifications can be detected *via* nanopore to questions of how, when, and where these modifications may be detected, as well as the threshold of methylation stoichiometry necessary for reliable detection (Gao et al., 2021; Leger et al., 2021; Pratanwanich et al., 2021). These questions are non-trivial and not limited to m6A, prompting the adaptation of modification-specific analysis workflows to other RNA and DNA modifications. For instance, the Novoa lab refined their EpiNano classifier (developed for m6A detection) to identify pseudouridine sites and quantify their stoichiometry in a software package called NanoRMS (Begik et al., 2021), and validated this approach for predicting pseudouridine sites *de novo*. Beyond estimates of modification stoichiometry, another emerging area is in the development of bioinformatic approaches to efficiently identify multiple types of nucleic acid modifications both within the same sample and along the same molecule. These approaches are expected to be of particular utility in the identification of sub-populations of differentially modified nucleic acids, as well as the study of “modification circuits” wherein ordered deposition of one modification may preclude or induce subsequent modification at another position (Spinelli et al., 1997; Arimbasseri et al., 2016; Han et al., 2017; Han and Phizicky, 2018). The Ares lab analyzed the correlation between modification status across multiple sites in full length budding yeast rRNAs at the single molecule level for 13 different RNA modifications, providing evidence that some modifications, particularly those within the catalytic core of the ribosome, are deposited in a coordinated manner (Bailey et al., 2022). Recent application of direct RNA sequencing to prokaryotic (Thomas et al., 2021) and eukaryotic (White et al., 2022) tRNAs is expected to yield further insight into the regulation of modifications on these highly modified molecules.

For DNA modifications, the long read lengths permitted by nanopore sequencing have also been applied to phased analysis, in which DNA methylations or other modifications of interest are identified as belonging to maternally- or paternally-inherited chromosomes (Gigante et al., 2019; Akbari et al., 2021). Such an approach can be used for genome-wide investigation of methylation patterns, as well as long-range changes in methylation patterns of relevance to the diagnosis and treatment of cancer (Nishiyama and Nakanishi, 2021). A recent report from Garg et al. used phased assembly data from nanopore sequencing to validate differential methylation associated with tandem repeat sequences, providing single molecule level support for a relationship between repeat copy number and CpG methylation in cis (Garg et al., 2021), and Flynn and others also evaluated the applicability of nanopore sequencing as a replacement for microarray profiling of methylomic variations associated with environmental exposures or disease phenotypes (Flynn et al., 2022). In addition, several groups have paired assessment of DNA methylation status with the interrogation of chromatin conformation, enabling the simultaneous read out of the spatial organization and modification of DNA (Ulahannan et al., 2019; Vermeulen et al., 2020). Finally, the relevance of both DNA and RNA methylation to differentiation and development is expected to motivate future developments in the application of nanopore modification detection to single cell biology, where already, proof of concept for pairing long read nanopore transcriptome sequencing with the 10x Genomics Chromium single cell platform (Lebrigand et al., 2020) has now been extended into an ONT-supported sequencing protocol and analysis pipeline.

## Challenges and limitations in modification mapping by nanopore sequencing

While advances in nanopore sequencing chemistry and base calling algorithms have provided rapid improvements in the accuracy of long read DNA and RNA sequencing, enabling higher sensitivity of modification detection, iterative refinements to nanopore sequencing technology can also complicate modification analysis. ONT’s continuous deployment approach to product development, as well as the non open source nature of both its sequencing chemistry and the Guppy base caller, has both positive and negative implications for modification analysis. On the positive side, improvements in base calling accuracy and the ability of base calling algorithms to identify nucleic acid modifications can enable future re-analysis of existing data, permitting higher resolution detection without the necessity of additional experiments. However, future changes to the nanopore sensor and/or motor protein chemistry, as well as software changes, have the potential to alter the type and/or magnitude of signal features, which could either enhance or blunt distinctions between nucleic acid modifications. Researchers should endeavor to re-evaluate modification signals after updates to sequencing chemistry or when re-base calling nanopore sequencing data using a new base calling algorithm.

**FIGURE 4:**
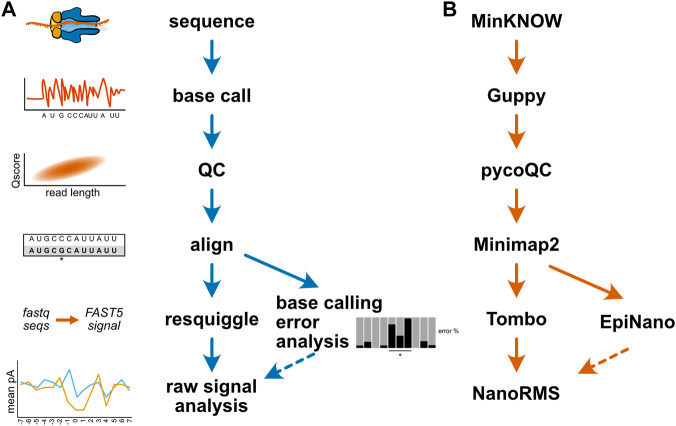
Example bioinformatic workflows for modification detection. **(A)** A generic diagram of the bioinformatic steps involved for mapping a nucleic acid modification. **(B)** An example analysis workflow for the detection of pseudouridine modifications in direct RNA sequencing. The MinKNOW software controls the sequencing instrument and permits real-time “fast” base calling with Guppy; however, for the purposes of modification identification, re-calling at higher accuracy after sequencing is recommended. The program pycoQC is one of several tools available for assessing run quality, and can be used to produce interactive plots of quality control metrics (Leger and Leonardi, 2019). Minimap2 is a pairwise alignment program optimized for long-read DNA or mRNA sequencing data, and can align direct RNA sequencing data in a splice aware fashion (Li, 2018). After alignment, the program EpiNano can be used to identify base calling “errors” consistent with pseudouridine modifications (Liu et al., 2019), and these sites can be further inspected at the single molecule level by resquiggling the data using Tombo (Stoiber et al., 2017) to permit analysis of raw signal features in regions of interest. The resquiggled data can be further analyzed with Tombo, or by using the software tool NanoRMS, which also enables prediction of pseudouridine modification stoichiometry (Begik et al., 2021).

Researchers new to long read sequencing are also faced with a dizzying array of software tools for both the preprocessing of nanopore data and the analysis of RNA and DNA modifications. The database long-read-tools.org currently catalogs over 500 nanopore sequencing tools across 35 categories (Amarasinghe et al., 2021). Table 2 details the software tools mentioned in this review. As alluded to above, changes to ONT’s sequencing hardware or software can render specific tools and algorithms obsolete in the absence of ongoing maintenance by individual developers, potentially making the selection of compatible software tools even more challenging over time. While a detailed comparison of current software options for the analysis of nucleic acid modifications is beyond the scope of this review, and has been ably outlined by others (Xu and Seki, 2020; Furlan et al., 2021; Wan et al., 2022), Figure 4A illustrates a generic bioinformatic pipeline for the detection of modifications not captured by existing base callers. Following sequencing, data should be base called using the highest accuracy mode available, followed by a QC step to assess read length, quality, and other run metrics, and to filter reads based on quality as needed. QCed and base called data can then be aligned to an appropriate reference sequence, enabling analysis of base calling errors, as well as the annotation of sequence and signal information (resquiggling) to permit analysis of raw nanopore signal. These two approaches can be complementary, as regions with strong differences in base calling error between an experimental and control sample may be further inspected to determine whether a candidate modification produces distortions at the level of raw current intensity and dwell time. Figure 4B provides one example workflow for the detection of pseudouridine modifications; however, after sequencing is complete, all remaining steps of this analysis have multiple software options available to choose from.

**TABLE 2:** Select software/bioinformatic tools mentioned in this text.

Name	Use	Description	Source	Additional documentation
MinKNOW	Sequencing	Operating software for ONT sequencing platforms	https://community.nanoporetech.com/downloads	
Guppy	Base calling	Current production basecaller from ONT	https://community.nanoporetech.com/downloads	https://denbi-nanopore-training-course.readthedocs.io/en/latest/basecalling/basecalling.html
Remora	Modified base calling	Bolt-on 5mC & other mod detection for ONT basecallers	https://github.com/nanoporetech/remora	
PycoQC	Quality control	Generate interactive QC plots for ONT sequencing data	https://github.com/a-slide/pycoQC	https://a-slide.github.io/pycoQC/
minimap2	Alignment	Long-read optimized pairwise aligner	https://github.com/lh3/minimap2	https://lh3.github.io/minimap2/minimap2.html
Nanopolish eventalign	Resquiggling/segmentation	Assigns raw signals to References sequence	https://github.com/jts/nanopolish	https://nanopolish.readthedocs.io/en/latest/quickstart_eventalign.html
Tombo resquiggle	Resquiggling/segmentation	Suite of ONT tools for modified base detection	https://github.com/nanoporetech/tombo	https://nanoporetech.github.io/tombo/index.html
ELIGOS	RNA modification detection	Base calling ‘error’ based modification prediction	https://gitlab.com/piroonj/eligos2	
Nanocompore	RNA modification detection	Modification detection from raw signal with paired samples	https://github.com/tleonardi/nanocompore	https://nanocompore.rna.rocks
EpiNano	m6A & other RNA mod. Detection	Base calling ‘error’ based modification prediction	https://github.com/novoalab/EpiNano	
NanoRMS	RNA modification stoichiometry	Raw signal + base calling error approach, validated on Ψ	https://github.com/novoalab/nanoRMS	

In ideal circumstances, researchers should expect to evaluate several different software tools and how well they perform for modification detection in any sufficiently new experimental and/or modification context; however, this may not be practical for all individuals or all situations. To address this, several groups have developed pipelines that permit more streamlined data analysis, and/or enable users to run several methods for detecting nucleic acids simultaneously. MasterofPores is a NextFlow pipeline developed by the Novoa lab that can both pre-process direct RNA sequencing data, and perform RNA modification detection using the tools Tombo and EpiNano (Cozzuto et al., 2020). The Snakemake pipeline MetaCompore enables RNA modification detection using six different software tools (Leger and Leonardi, 2021), and the METEORE pipeline takes an analogous approach to DNA methylation detection (Yuen et al., 2021), enabling users to run multiple analyses simultaneously and compare their outputs. While systematic benchmarking of modification detection tools remains limited, these and other reports suggest that selecting the appropriate software often involves tradeoffs between sensitivity, specificity, and accuracy of *de novo* modification prediction.

Finally, while the generation of long read sequence and signal information opens exciting opportunities for the study of nucleic acid modifications at the single molecule level, identifying novel modifications by nanopore remains computationally intensive. Multiple steps of nanopore sequencing analysis have been optimized for faster processing on graphics processing units (GPUs), including base calling (Teng et al., 2018), alignment, re-squiggling, and modification calling (Cozzuto et al., 2020; Gamaarachchi et al., 2020). Although the MinKNOW software can perform rapid base calling in real time, for the purposes of modification detection, it is recommended to re-base call all sequencing data using the highest accuracy model available in order to distinguish between genuine modification signal and sequencing errors; however, higher accuracy base calling comes with higher computational costs. In addition, analysis of raw nanopore signal can also be resource intensive, making it generally intractable to analyze per-read raw signals across large windows of sequence. Instead, researchers may be best served by evaluating mean current intensity and/or dwell at the genome- or transcriptome-wide level, and then if needed, examining signals of interest over a much smaller (15–30 nt) window (Begik et al., 2021; Stephenson et al., 2022; White et al., 2022). Alternatively, raw per-read information can be collapsed down to a binary evaluation of whether individual nucleotides are modified or unmodified, facilitating interrogation of much larger regions at the single molecule level (Bailey et al., 2022).

## Concluding remarks

More than 41 naturally occurring DNA modifications (Sood et al., 2019) and 170 RNA modifications (Boccaletto et al., 2022) have been identified in nucleic acids to date, the majority of which lack high throughput sequencing detection methods. While detection of cytosine and adenine nucleobase methylations have been the focus of intensive methodological development, the studies outlined in this review demonstrate that nanopore sequencing is already being actively used in many experimental contexts beyond the mapping of m6A and 5 mC modifications. These and other experiments have paved the way for more detailed examination of the entire landscape of modifications using nanopore sequencing. Improvements in third generation sequencing accuracy, computational methods, and the generation of additional synthetic and biological training data for modified bases are expanding the alphabet of canonical and modified nucleotides that can be directly identified by nanopore sequencing. Together, these developments will enable new insights into how, when, and where nucleic acid modifications are deposited, maintained, removed, and regulated.
